# Factors influencing specialist care referral of multidrug- and extensively drug-resistant tuberculosis patients in Gauteng/South Africa: a descriptive questionnaire-based study

**DOI:** 10.1186/1472-6963-13-268

**Published:** 2013-07-09

**Authors:** Deliwe Nkosi, Saskia Janssen, Xavier Padanilam, Rianna Louw, Colin N Menezes, Martin P Grobusch

**Affiliations:** 1 South African Field Epidemiology and Laboratory Training Programme (SAFELTP), Johannesburg, South Africa; 2 University of Pretoria, Pretoria, South Africa; 3 Sizwe Tropical Diseases Hospital, Sandringham, Johannesburg, South Africa; 4 Department of Infectious Diseases, Division of Medicine, Center of Tropical Medicine and Travel Medicine, Amsterdam Medical Center, University of Amsterdam, Meibergdreef 9, PO Box 22660, 1100 DD Amsterdam, The Netherlands; 5 Department of Medicine, Division of Infectious Diseases, Faculty of Health Sciences, University of the Witwatersrand, Johannesburg, South Africa; 6 Institute of Tropical Medicine, University of Tübingen, Tübingen, Germany

**Keywords:** Tuberculosis, Drug-resistance, Delay of referral, South Africa

## Abstract

**Background:**

Sizwe Tropical Diseases Hospital is the only specialized Hospital for the management of multidrug-resistant (MDR)-TB and extensively drug-resistant (XDR)-TB cases in Gauteng Province. In South Africa, there is a mismatch between numbers of individuals with a laboratory diagnosis of drug-resistant tuberculosis (TB) and those being referred for the initiation of specialist treatment. We determined reasons for non-referral of MDR-TB and XDR-TB cases.

**Methods:**

We conducted a descriptive questionnaire-based study amongst provincial primary health care facilities (PHC) and hospitals providing routine care for (drug-susceptible) TB, regarding specialist care referral of patients whose TB culture and susceptibility testing confirmed MDR-TB or XDR-TB diagnoses in the first half of 2008.

**Results:**

In total 148 cases were analyzed; 144/148 (97%) had MDR-TB and 4/148 (3%) had XDR-TB. The main reason for non-referral to specialist care was loss to follow up, for patients diagnosed in-hospital (74/97; 76%) as well as in PHCs (11/21; 52%). Nineteen per cent (18/97) of patients diagnosed in hospital versus 33% (7/21) of patients diagnosed in PHCs deceased before referral.

**Conclusions:**

A significant problem in the fight to control DR-TB is follow-up after diagnosis with a delay in patient tracing. TB Focal Points in hospital need to be strengthened in order to improve on patient follow-up and care, and tracer teams should assist with community follow up.

## Background

Multidrug- and higher degrees of drug resistant *Mycobacterium tuberculosis* have become a global public health issue of high priority. According to the World Health Organization (WHO) 2008 estimates, there were 440.000 cases and 150.000 deaths of multidrug-resistant tuberculosis (MDR-TB) globally and only about 1% of these cases were on treatment regimens based on WHO recommended standards [1]. In 2010, extensively drug-resistant (XDR)-TB was reported in 58 countries throughout all regions of the world. Due to the emergence of MDR-TB, the WHO developed a directly observed therapy short-course (DOTS) Plus strategy in 2000 [2]. This strategy aims at ensuring correct identification and proper management of MDR-TB patients. DOTS-Plus treatment of MDR-TB cases has been proven to be highly cost effective in certain areas [3,4].

Treatment delays have been attributed to various factors, such as living far from the health care facility, feeling a high degree of stigma, seeking initial care at a non-professional health care facility and having more than one health care encounter before diagnosis. However, health providers’ and health systems’ inherent delays have been found to account for the major part of the total delay [5,6]. The delay in the diagnosis and treatment of MDR-TB can result in patients developing persistent disease, progressive parenchymal destruction, higher bacillary loads, continuing transmission and increased mortality. In most public health settings there is lack of adequate and appropriate infection-control measures and, together with high human immune-deficiency virus (HIV) co-infection rates in certain settings [7], this represents a public health emergency, calling for earlier detection and treatment of drug resistant TB [8].

South Africa currently ranks fourth amongst countries with a high absolute number of MDR-TB cases, with an estimated number of 13.000 (6.7% of retreatment and 1.8% of new TB cases) [1,5,6], and with a high rate of HIV co-infection [7]. Comprehensive programmatic management of patients with MDR-TB became national South African policy in 2000 and was implemented through provincial MDR-TB referral centres [9]. The most advantageous strategy for MDR-TB patients capitalizes on early diagnosis of MDR-TB [10].

Gauteng Province with only 1.4% of South Africa’s land surface is highly urbanized and has an estimated population of between 8.8 and 9.5 million people. As South Africa’s economic hub, Gauteng attracts people from all across the country as well as the Southern African region who come in search of employment opportunities. The province is divided into six health care districts with a number of hospitals and PHCs. Within the larger hospitals, TB Focal Points were established in most of the major hospitals. TB Focal Points are centres dedicated to effective diagnosis, investigation, and treatment of TB and simultaneous investigation for HIV co-infection. Usually jointly staffed by doctors and PHC nurses, they assist in the diagnosis and referral of drug resistant TB. If patients are diagnosed with MDR- or XDR-TB, they are transferred for treatment to Sizwe Tropical Diseases Hospital (SH), a 268-bed specialized treatment centre for MDR- and XDR-TB patients.

According to the 2008 South African guidelines on management of MDR-TB, MDR-TB patients were admitted for at least the first six months or until they had produced two consecutive monthly culture-negative sputa [11].

Prior to this study, SH estimated that 30-40% of culture-confirmed patients were unaccounted for in terms of treatment in Gauteng province.

The objectives of the study were to determine the number of culture and DST confirmed MDR- and XDR-TB cases in Gauteng that were not referred for specialist treatment at SH during the study period, and to identify reasons for this, with a focus on the functioning of health care facility follow up systems.

## Methods

### Study design

A descriptive study was conducted from October 2008 to December 2008, comprising retrospective review of patient records and laboratory data of all patients, whose TB culture and susceptibility testing confirmed MDR- or XDR-TB in the first half of 2008. Patients who were suspected of having DR-TB at any of the health care facilities throughout Gauteng province had their drug susceptibility testing (DST) performed at the South African National TB Reference Laboratory (NTBRL). The DST results were sent back from the NTBRL to the requesting health care facilities; drug-resistant results indicating to the facilities the eligibility of the patients for being referred to SH for specialist treatment. A case was defined as a new laboratory-confirmed MDR- or XDR-TB patient who was not on treatment for DR-TB, identified from the NTBRL database between 1 January 2008 and 30 June 2008. Using a convenience sample, 40 health care facilities (hospitals and PHCs) in four of the six districts of Gauteng province were identified. Health care professionals at these institutions were interviewed using semi-structured questionnaires with regard to their patient referral procedures in general; and on those patients whose referral failed in particular.

### Data collection

Data of all patients who were admitted to SH for initiation of DR-TB treatment between January and June 2008 were compared to NTBRL data of all the laboratory-confirmed MDR- and XDR-TB cases, which were diagnosed from 1 January 2008 to 30 June 2008. Patients who were positive for MDR- or XDR-TB but did not appear on the SH admission record sheet were traced back to referral facilities. Information on referral health care facility and date of laboratory test confirmation was collected from the NTBRL data. A site manager or a professional nurse at each identified facility was interviewed using a semi-structured questionnaire after signing a consent form, with regard to (part A) specificities of site management of newly identified MDR- and XDR-TB cases, and (part B) on those patients not subsequently appearing at SH.

### Statistical analysis

From the various health facilities, data on health care workers’ responses to the questionnaires was categorized and reported as percentages and absolute frequencies. Continuous variables were reported in median and range. All data was analyzed using Epi Info version 3.5.1, 2008 (CDC, Atlanta, GA).

### Ethical approval

The study was approved by the ethics committees of the University of the Witwatersrand, Johannesburg, and the University of Pretoria.

## Results

Four out of six districts (Johannesburg, Tshwane, Sedibeng, Metsweding) contributed data. A total of 148 cases in 40 facilities (29 PHC and 11 hospitals) were analyzed. One hundred and forty-four cases (97%) were MDR-TB and 4 (3%) were XDR-TB. The median age in files specifying age (95/148) was 35 years (range: 10–82 years) and amongst those data sets specifying patient sex (121/148), there were more females (59%; 72/121) than males.

Reasons for failure of referral to specialist care of MDR- and XDR-TB patients are shown in Table 1. One hundred and eighteen cases (80%) of the total number of cases identified in the NRTBL were not seen back for results in the diagnosis-initiating health care facilities, and could therefore not been referred for treatment at SH. The most important reason for non-referral to specialist care was loss to follow up, for patients diagnosed in-hospital (74/97; 76%) as well as in PHCs (11/21; 52%). There was a high mortality during the interval between diagnosis and referral; of the patients diagnosed in hospital, 19% (18/97) deceased before referral versus 33% (7/21) of patients diagnosed in PHCs, resulting in an overall mortality of 21% (25/118).

**Table 1:** Reasons for cases not being seen for results in facilities, Gauteng, Jan 2008 - June 2008

**Reason**	**Hospital n (%)**	**Clinic n (%)**	**Total count**
Loss to follow up^1^	74 (76%)	11 (52%)	85 (72%)
Deceased	18 (19%)	7 (33%)	25 (21%)
Did not come for results^2^	4 (4%)	1 (5%)	5 (4%)
Relocated	1 (1%)	2 (10%)	3 (3%)
Total	97(100%)	21 (100%)	118 (100%)

^1^Tracing of patient by health facility worker unsuccessful.

^2^Patients who were traced e.g. by phone (and had appointments) who did fail to present at the health facility for follow-up.

Looking into obstacles to refer patients correctly; one major problem was that of health care facility staff interviewed, 24/29 in PHC (86%) and 7/11 in hospitals (64%) were not aware of the national MDR-TB guidelines (Table 2). When comparing methods of results delivery from the laboratory to the clinical care personnel in PHC and hospitals (Table 3), about 96% (28/29) of PHC used a courier service, whilst 90% (10/11) of hospitals used fax services. The majority of PHCs (18/29; 62%) arrange for home visits to inform the patients of the outcome of their results whilst most hospitals (7/11; 63%) use phone calls. Most PHC (16/29; 55%) routinely send patients with referral letters and an escort when referring them to SH for treatment, whilst only 4/11 hospitals (36%) did the same. After transfer, only 1/29 PHC and none of the hospitals routinely follows up on referred cases to ensure that the patient has reached SH (Table 3).

**Table 2:** Factors that affect implementation of MDR-TB guidelines in referring facilities

**Factor**	**Hospital n/N (%)**	**Clinic n/N (%)**
Staff not aware of MDR-TB guidelines	7/11 (64%)	24/29 (83%)
Staff do not know SH telephone number	4/11 (36%)	11/29 (38%)
Patient follow up perceived as difficult	11/11 (100%)	29/29 (100%)

**Table 3:** Referral system of cases in clinics and hospitals

	**Hospital**	**n/N (%)**	**Clinic**	**n/N (%)**
Mode of results delivery to facility	Fax	10/11 (91%)	Fax	1/29 (3%)
Method used by facility to inform patients about results	Home visits	4/11 (36%)	Home visits	18/29 (62%)
Method used by facility to inform SH	Phone	7/11 (64%)	Phone	13/29 (45%)
Method used by facility to send patient to SH	Referral letter and escort	4/11 (36%)	Referral letter and escort	16/29 (55%)
Follow up of patients by facility after transfer to SH	Never	4/11 (36%)	Never	4/29 (14%)

## Discussion

In this study, loss to follow up was the most important reason for failure of referral of MDR- and XDR-TB patients to specialist care, both for hospital and PHC diagnosed cases. A considerable number of patients deceased in the interval between diagnosis and referral, highlighting the importance of a stringent, timely and effective referral system for this extremely vulnerable patient group. Most operational problems mentioned, such as the lack of knowledge of MDR-TB guidelines or communication procedures regarding referral, seem to be rectifiable with a reasonable amount of effort and cost.

At the time this study was carried out, referral of newly diagnosed TB patients with multi- or higher degrees of drug resistance to specialized units for treatment until repeated culture negativity was managed according to the by then actual national guidelines in South Africa. In the light of non-feasibility in many parts of the country and assumedly in other settings worldwide, with an evident mismatch between beds available and patient numbers, guidelines are now moving towards favoring outpatient care. Nevertheless, an outpatient-based care system will require a sound public health system to capitalize on [11,12].

In this study, absolute numbers of MDR- and XDR-TB patients who were not started on appropriate treatment (because they were not referred) were highest for those diagnosed in hospitals (compared to those diagnosed at clinics). This is in part due to the fact that even though the laboratories in these hospitals have documentation of the sputum culture results, the personnel at the TB focal points did not have any documentation of having seen these cases after they were discharged. The cases are discharged and not routinely provided with a date to come back for the TB results. Some of these patients died before results could be communicated back to them. In Africa, death and default rates due to TB are high and they are linked to high rates of HIV co-infection and weak health care services [10]. Immune status, the application of appropriate drug treatment, culturing of mycobacteria within 30 days and age are significant factors associated with survival in MDR-TB cases [13].

The hospitals have the convenience of receiving their results via fax since the laboratory is located on the hospital premises. In the clinics, a courier service is utilized which delivers the results daily. The clinics task is to ensure that cases are informed of their laboratory results by sending DOT supporters/community health workers to do home visits and providing an escort who will make sure that they reach the referral hospital.

One possible explanation for referral problems occurring on staff level is that there may be a considerable level of staff rotating through different health center rather than “TB staff” being permanently in their positions; to what extent this is a contributing factor would remain to be elucidated.

Our study demonstrates that there is room for improvement at the level of the referring health care facilities. Clear allocation of responsibilities and training of staff identified to carry out this important task is paramount for sustainable improvement.

Our study has various limitations. The sample size was limited and data were missing in some of the patient records for sex, age, occupation and type of residence. The role of laboratory delays in terms of time lapse between a positive result and its communication back to the specimen-submitting unit was not studied because there were no data available on the date of sputum arrival in the healthcare facility.

## Conclusions

Dysfunctional health systems will result in new anti-TB drugs following the same path to resistance that the current drugs have taken [14]. For efficient treatment, patients have to be identified, traced and diagnosed before they can be referred for treatment, no matter whether treatment will be in- or outpatient based.

Our findings suggest that the main reason for non-referral of MDR- and XDR-TB to specialized treatment facilities is loss to follow up, mainly due to a lack of well-functioning follow-up and referral systems in hospitals and clinics diagnosing DR-TB. To tackle the deficits in patient follow-up and care after diagnosis (as an important reason for the lack of continuation of care for MDR- and XDR-TB patients), TB referral systems (capitalizing on the existing TB focal point system) need to be strengthened.

## Abbreviations

DOTS: Directly Observed Therapy – Short-course; HIV: Human Immunodeficiency Virus; MDR-TB: Multi-Drug-Resistant Tuberculosis; NTBRL: National Tuberculosis Reference Laboratory; PHC: Primary Health Care facility; SH: Sizwe Tropical Diseases Hospital; TB: Tuberculosis; WHO: World Health Organization; XDR-TB: Extensively Drug-Resistant Tuberculosis.

## Competing interests

None of the authors has any competing interest to declare.

## Authors’ contributions

DN carried out the field work and wrote the first draft of the paper. SJ contributed to data interpretation, the compilation of background information and writing of the paper. XP, RL and CNM contributed to phrasing the study question, data interpretation and writing of the paper. MPG conceived the study, oversaw the conduct of the study and contributed to all stages of the paper writing. All authors have contributed to the writing and approval of the final version of the paper.

## Authors’ informations

At the time of the study, MPG was Professor of Infectious Diseases at the University of the Witwatersrand, Johannesburg, and together with XP and RL responsible for the transformation of Sizwe Tropical Diseases Hospital to a hospital specialized in the treatment of MDR- and XDR-TB.

## Pre-publication history

The pre-publication history for this paper can be accessed here:

http://www.biomedcentral.com/1472-6963/13/268/prepub
